# Optimizing the input feature sets and machine learning algorithms for reliable and accurate estimation of continuous, cuffless blood pressure

**DOI:** 10.1038/s41598-023-34677-9

**Published:** 2023-05-12

**Authors:** Rajesh S. Kasbekar, Songbai Ji, Edward A. Clancy, Anita Goel

**Affiliations:** 1 Department of Biomedical Engineering, Worcester Polytechnic Institute (WPI), Worcester, MA USA; 2 Department of Electrical and Computer Engineering, Worcester Polytechnic Institute (WPI), Worcester, MA USA; 3 Nanobiosym Research Institute, Nanobiosym, Inc. and Department of Physics, Harvard University, Cambridge, MA USA

**Keywords:** Machine learning, Predictive medicine, Hypertension

## Abstract

The advent of mobile devices, wearables and digital healthcare has unleashed a demand for accurate, reliable, and non-interventional ways to measure continuous blood pressure (BP). Many consumer products claim to measure BP with a cuffless device, but their lack of accuracy and reliability limit clinical adoption. Here, we demonstrate how multimodal feature datasets, comprising: (i) pulse arrival time (PAT); (ii) pulse wave morphology (PWM), and (iii) demographic data, can be combined with optimized Machine Learning (ML) algorithms to estimate Systolic BP (SBP), Diastolic BP (DBP) and Mean Arterial Pressure (MAP) within a 5 mmHg bias of the gold standard Intra-Arterial BP, well within the acceptable limits of the IEC/ANSI 80601-2-30 (2018) standard. Furthermore, DBP’s calculated using 126 datasets collected from 31 hemodynamically compromised patients had a standard deviation within 8 mmHg, while SBP’s and MAP’s exceeded these limits. Using ANOVA and Levene’s test for error means and standard deviations, we found significant differences in the various ML algorithms but found no significant differences amongst the multimodal feature datasets. Optimized ML algorithms and key multimodal features obtained from larger real-world data (RWD) sets could enable more reliable and accurate estimation of continuous BP in cuffless devices, accelerating wider clinical adoption.

## Introduction

There is an increasing need for noninvasive, continuous and cuffless approaches for BP measurement that minimize patient discomfort. Moreover, continuous BP monitoring has significant advantages compared to sporadic or intermittent clinical BP measurements taken in an office setting or at home. The advent of mobile devices, and wearables has unleashed a demand for continuous monitoring of BP using cuffless sensors integrated with smart-phone-based applications. Furthermore, BP telemonitoring, which involves self-measurement of BP and transmission of the data to the patient’s physician, has been shown to be more efficacious at lowering SBP and DBP than traditional office-based care^1^, enabling the collection of larger RWD datasets that can be used to further enhance efficacy of measurement and clinical decision making.

Despite many technical approaches and consumer products on the market that claim to measure BP via a cuffless device, there remain many significant barriers to clinical adoption. These include problems in accuracy, reliability, lack of optimized machine learning algorithms, and the need for periodic calibrations against a conventional cuff-based blood pressure measurement. In fact, the Aurora project^2,3^, conducted by Microsoft Research and one of the most comprehensive and important studies on assessing the accuracy of such cuffless BP measurement devices, concluded that the current generation of cuffless devices with their various regression models provide no additional value in measuring resting auscultatory or 24-h ambulatory cuff BP when compared with a baseline model in which BP was predicted without an actual measurement^2,3^. The European Society of Hypertension consensus document^4^ has concluded that cuffless BP devices have considerable potential for changing the diagnosis and management of hypertension; however fundamental problems regarding the reliability and accuracy of such BP measurement devices have limited widespread clinical usage. The authors further conclude that the question of ‘trusting the measurement’^4,5^ in the individual patient will be the most important issue to be addressed by future development in technology and clinical validation.

The accurate and reliable estimation of BP has been difficult as a result of challenges with extracting the correct feature data sets from measurements of continuous BP in a cuffless device using pulse arrival time (PAT) or pulse wave morphology (PWM) due to a lack of consistent theoretical understanding and confounding physiology^5^. For example, BP and photoplethysmography (PPG) waveforms can be affected by many factors such as pre-ejection period (PEP), smooth muscle contraction, hormonal changes, metabolic activity, cardiovascular disease, etc. Moreover, previous approaches to cuffless BP measurement devices have relied primarily on regression or singular machine learning algorithms to analyze feature datasets. Such publications built models that relied mostly on data from healthy patients. The clinical usage of of these methods depends on how applicable they are to hemodynamically compromised patients.

Our motivation for this paper, therefore, was twofold: (i) to identify a comprehensive set of critical features that impact the estimation of BP based on a strong theoretical framework, and (ii) compare the use of several machine learning algorithms statistically on a real-world (RWD) dataset and show the importance of choosing the most optimal one to assess the accuracy of estimating blood pressure in a cuffless device. Herein, we demonstrate a method for estimating SBP and DBP, using extracted feature datasets from PPG signals along with other demographic data from hemodynamically compromised subjects. We also compare the performance of four classical machine learning (ML) algorithms and one deep learning algorithm to estimate the SBP, DBP and MAP using intra-arterial BP as the gold standard label for our supervised learning ML algorithms. We also tested for statistically significant differences in error means and standard deviations using the various feature datasets and the various ML algorithms. We present a theoretical framework and demonstrate how proper selection of optimized ML algorithms used on larger, impactful multimodal feature datasets could enable reliable and accurate continuous BP estimation for earlier detection and better management of hypertension.

For routine monitoring, diagnosis and treatment, arterial BP is considered a universal indicator of hypertension and cardiovascular health. The gold standard for BP measurements in hospital and professional settings is intra-arterial catheterization, a method that is invasive and requires a sensor such as a strain gage to be in fluid contact with blood at an arterial site^6^. The noninvasive method used in clinics is the auscultation-based BP cuff with mercury manometer method^7,8^, where the brachial artery is occluded with a cuff placed around the upper arm inflated above the SBP, and Korotkoff sounds that are detected using a stethescope during cuff deflation. Both of these methods have been used as a gold standard for over 100 years with little innovation. However, patient discomfort, reduced patient mobility and intermittent monitoring are some of the limitations of this methodology. In addition, the effects of posture, body position, white coat hypertension and cuff size significantly affect the accuracy of the readings^9^, making this an unreliable method for measuring BP.

Automated cuff-based methods, whether used in the clinic or at home, have an inherent accuracy requirement of ± 5 mmHg bias and ± 8 mmHg standard deviation as a key criterion in this standard^10^. Wearing a cuff can be uncomfortable for elderly, diseased or handicapped subjects. It has resulted in dizziness and fainting in some instances. To address this issue and increase user friendliness, more comfortable cuffs for the wrist have been developed. These devices, however, have significantly lower measurement accuracy than upper arm BP monitors^11^.

There are three measures of BP that are powerful predictors of hypertension: average or true level, diurnal variation and short-term variability^12^. Most clinical and epidemiologic data are only available for the average or true level using intermittent cuff based BP measurements. However, continuous BP monitoring using cuffless devices could help elucidate the correlations between diurnal variation and short-term BP variability and various diseases, enabling earlier diagnosis and better management of diseases such as chronic renal failure, malignant and secondary hypertension, pre-eclampsia and autonomic neuropathy^9^. For instance, cardiovascular morbidity and mortality have already been shown to be more correlated with nighttime BP than daytime BP^9^. Increased ambulatory BP variability has also been shown to be correlated with the development of early carotid arteriosclerosis and a high rate of cardiovascular morbidity^9^. Hence, continuous BP monitoring has significant advantages compared to intermittent BP taken in a clinic or at home (Supplementary Information).

Continuous BP is typically estimated using features extracted from (i) use of Pulse arrival time (PAT) or Pulse transit time (PTT) and (ii) use of Pulse wave morphology (PWM).

### Pulse arrival time (PAT) or pulse transit time (PTT) in BP estimation

BP regulation in arterial vessels is a complex phenomenon. It is affected by many variables, including blood viscosity, stiffness and cross-sectional area of the blood vessels, and hormonal changes resulting in smooth muscle contraction. There is, however, a strong association between BP and the velocity of the pulse wave that propagates through the arterial network. This relationship is given in its simplest form by the Moens-Korteweg equation:

1 $$BP=K\_1\cdot \mathrm{log}[(1/\mathrm{PWV})+\mathrm{K}\_2 ]$$

where $K\_1$ and $K\_2$ are constants and PWV is the pulse wave velocity.

The relationship can be further modified^13^ based on the age of the patient, the arterial wall area and arterial compliance. In practice, the approach to the noninvasive continuous estimation of SBP and DBP is based on the measurement of the PAT instead of the PTT when measured using the ECG R-wave and the pulse peak at the distal end. The PAT is the time delay between the ECG R-wave and the arrival of the pulse peak at a distal location, such as the wrist. In contrast, the time delay between the pulse peaks at a proximal location to its arrival at a distal location, such as the wrist, is defined as the PTT. If PAT is measured using a location just distal to the heart, it includes the pre-ejection period (PEP)—the time associated with the ejection of blood from the heart into the aorta^14^. The PEP can vary with BP and intra- or intersubjective variation; therefore, PAT is considered an unreliable predictor of SBP^15,16^. PAT and PTT are inversely related to pulse wave velocity if the distance between the proximal and distal measurement points is maintained constant. PAT or PTT have become well-established physiological correlates of SBP, DBP, and MAP.

Several researchers have developed continuous, cuffless BP estimation methods using PAT or PTT^17–22^ based on a peripheral PPG sensor. The second sensor may be placed at the sternum and timing acquired from ECG using ECG electrodes, or another peripheral site and timing acquired from PPG, seismocardiogram using an optical sensor array, or ballistocardiogram using integrated strain gages and a reflective optical sensor array. Hall Effect sensors, bioimpedance using an impedance electrode array or pressure tomography waveforms have also been used as substitutes for the PPG signal.

### Pulse wave morphology (PWM) and feature extraction in BP estimation

While PTT is a good predictor of BP, it also varies with arterial compliance and cross-sectional area, which can change with time. These changes are confounding factors in the accurate estimation of BP. BP is better characterized by the cardiac hemodynamic Windkessel model^23^, a first-order differential equation relating arterial compliance, resistance, BP and cardiac output or blood flow.

Nonetheless, the pulse wave contour of the PPG signal carries important information related to this dynamically changing equation and is feature-rich in estimating arterial BP. Therefore, the extraction of features from this distal PPG waveform can be used to estimate SBP, DBP and MAP and has been used effectively by some investigators^24^ to predict BP. Lee et al.^19^ used a Hall Effect sensor to record a pulse waveform and used a similar multifactor model to estimate SBP, DBP and MAP. Their bias error was approximately 8–12 mmHg—above the threshold for clinical acceptance.

Various ML algorithms^13,14^ using machine and deep learning techniques have been utilized on measures associated with SBP, DBP, and MAP, such as PPG, bioimpedance, pressure tomography or other waveforms, pulse rate, and other demographic data. Liu et al.^24^ used a multidimensional regression model on ten subjects to estimate BP from PTT, ejection time ratio, heart rate, estimated arterial blood volume and volume change during systolic and diastolic cycles. They found that in addition to PTT, using the best few parameters resulted in lower errors than using all the parameters. The bias error averaged 7–8 mmHg for SBP and 4–5 mmHg for DBP. Pielmus et al.^25^ used the morphology of PPG, bioimpedance and arterial tomography signals to estimate BP from beat-to-beat spectral features using a polynomial model. Each biosignal performed similarly, with mean absolute errors from 4.64 to 8.86 mmHg and standard deviations from 4.67 to 9.48 mmHg. Models based on arterial tomography were the least consistent. Limitations of the study included hemodynamic variability, noise and a small sample size of 10 subjects.

Schlesinger et al.^26^ estimated SBP and DBP using a PPG sensor and a Siamese twin convolution neural network deep learning method and found mean errors for SBP, MAP and a DBP of 7.98, 5.51 and 4.11 mmHg, respectively. Ruiz-Rodriguez et al.^27^ used a deep belief network with a restricted Boltzmann machine to train over 572 subjects, of which 525 were assigned to the training cohort and 47 to the testing cohort. The mean prediction biases were − 2.98 ± 19.35, − 3.38 ± 10.35, and − 3.65 ± 8.69 mmHg for SBP, MAP, and DBP, respectively. These were unsuitable for clinical application due to the broad limits of agreement. Daxin et al.^28^ used a deep recurrent neural network to estimate BP using sliding window sampling. The sliding window captured the time dependence among continuous heartbeats separated by sampling segmentation. By carrying out experiments on a dataset of 119 subjects, the authors demonstrated that this strategy could effectively improve the accuracy of BP estimations. The systolic and diastolic errors were 5.73 mmHg for SBP and 3.50 mmHg for DBP. The mean arterial deviation was also in the 3 to 4 mmHg range. A recurrent neural network^29^ based on a nonlinear autoregressive model with 18-time delays on the ECG and the PPG signal and two-time delays on the BP signal was used to estimate BP in three patients using static and readings obtained with the use of exercise maneuvers. The model's accuracy on the estimated DBP met the IEC 80601-2-30^10^ standard protocol requirement; however, the SBP estimation was slightly large. Jeong et al.^30^ used a combined deep CNN-LSTM network-based multitasking learning architecture for continuous monitoring, however their model was tested on only ten subjects and did not include verification of generalizability of the model.

## Methods

### Ethical statement

Approval was granted by the Ethics Committee^31,32^ (CCI—Committee on Clinical Investigations—protocol #2001P001699) of Beth Israel Deaconess Medical Center for the hemodynamically compromised dataset. The CCI is charged with safeguarding the rights and welfare of human subjects by making determinations regarding ethical standards and evaluating the risk and benefit ratio of all studies. For the healthy dataset, protocol # CP-B150X approval was granted by the Alpha Independent Review Board (Registration #IRB00006205). Alpha Independent Review Board is fully accredited by the Association for the Accreditation of Human Research Protection Programs (AAHRPP). Research involving human research participants was performed in accordance with the Declaration of Helsinki. Informed consent was obtained from all participants and/or their legal guardians. All research was performed in accordance with relevant guidelines and regulations.

### Experimental protocol

#### Data collection and feature extraction in healthy subjects

**Figure 1:**
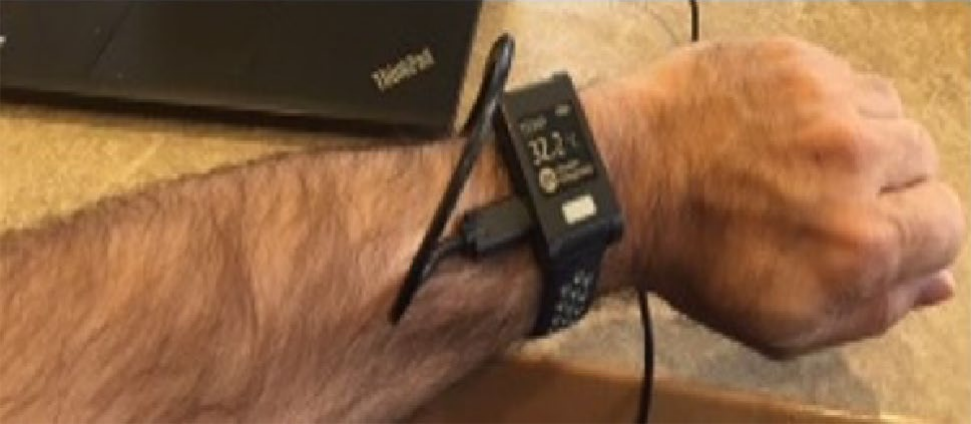
Experimental setup using the Maxim Watch (healthy patient dataset).

Our first set of experiments was used to show the effectiveness of our methods on 23 healthy subjects (see Table 1 for subject demographics). Reference BP and pulse rate were taken with a mercury sphygmomanometer (ADC 922-10SABK Diagnostix Traditional Desktop Mercury Sphygmomanometer Small Adult) and a stethoscope (Classic II S.E. Teaching 40" Stethoscope). Two separate trained staff took these measurements such that there was consensus following the IEC standard for BP measurement (IEC 80601-2-30 Standard for B.P. monitoring^10^, 2018).  We measured the PPG signals for healthy patients using a Maxim watch (Maxim Integrated Inc., CA, model MAXREFDES103). The Maxim Watch used red and infrared PPG sensors (separated by 5 mm) to derive PTT and other features. Red and infrared PPG waveforms (sampled at 400 Hz with 19-bit resolution) from the pair of sensors separated by 5 mm were collected noninvasively over the radial artery on the left wrist of each subject. The subjects sat upright for the measurements, which took approximately 5 min. Figure 1 shows the experimental setup for data collection. The distal sensor pair was located over the radial artery, approximately 2 cm from the wrist crease. For BP estimation, we extracted PTT (over the 5 mm distance), pulse rate, and five demographic factors (waist size, weight, sex, temperature, age)—for seven input features.

**Table 1:** Healthy and hemodynamically compromised subject demographics.

Descriptor	Healthy subjects (n = 23)	MIMIC I hemodynamically compromised datasets (n = 126)
Male	12 (52%)	75 (60%)
Female	11 (48%)	51 (40%)
Age (> 40 years)	12 (52%)	116 (92%)
Age (< 40 years)	11 (48%)	10 (8%)

**Figure 2:**
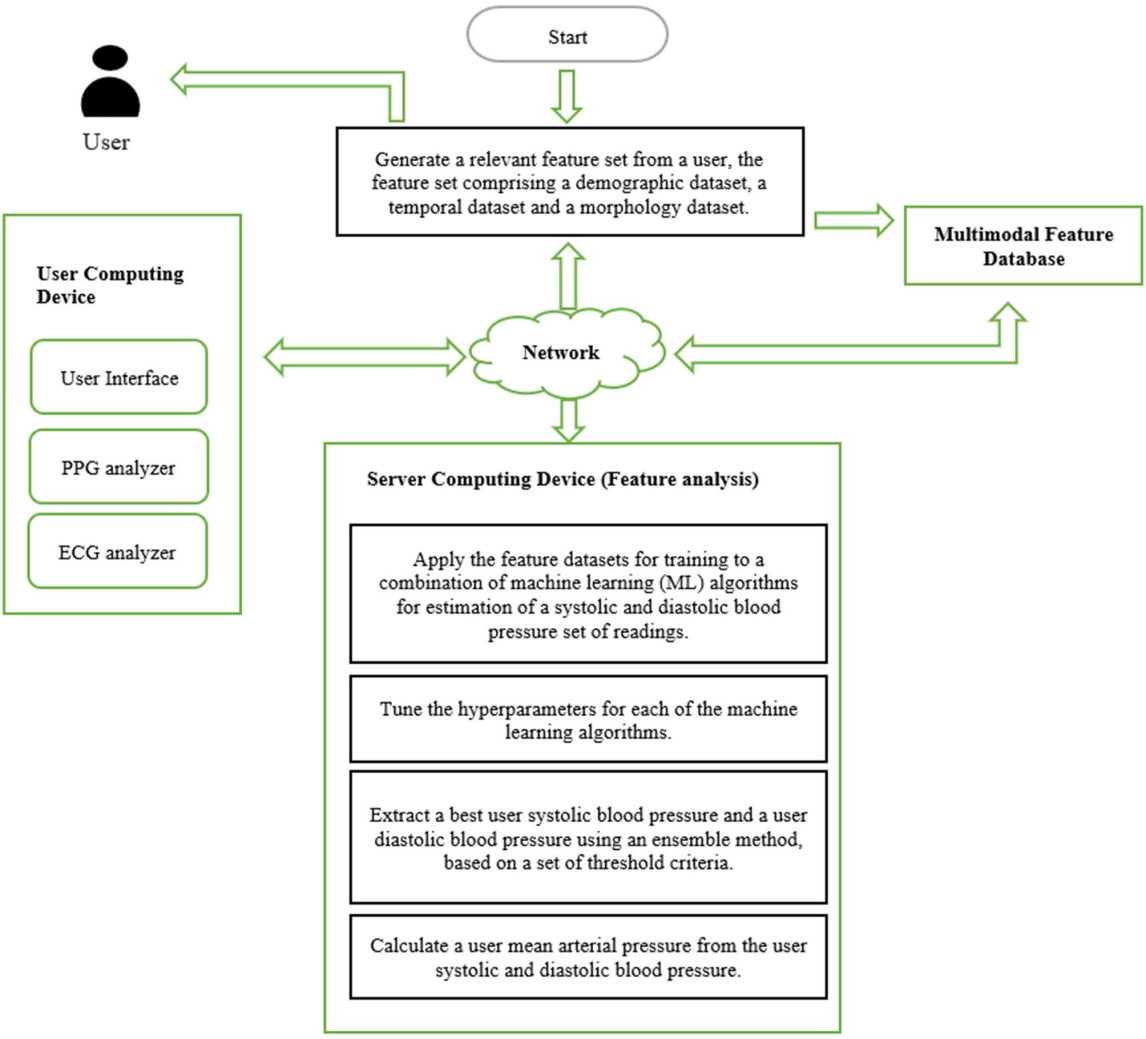
Schematic diagram of the proposed methodology.

We used MATLAB to analyze PPG signals and demographic data for healthy and hemodynamically compromised patients. The goal of estimating BP in healthy patients was to show feasibility. For *hemodynamically compromised data*, we extracted features from demographic, measurement and waveform data for PPG and ECG-R waves from 90 ICU patients in the Physionet^31^ Multi-parameter Intelligent Monitoring for Intensive Care or MIMIC I database^32^. The features were then used in five ML models to estimate BP. Figure 2 shows a schematic of the data flow, the associated hardware as well as the algorithmic steps involved in the proposed methodology.

### Patient data and feature extraction in hemodynamically compromised patients

The Physionet^31^ Multi-Parameter Intelligent Monitoring for Intensive Care or MIMIC I database^32^ consists of 90 patient recordings, each typically containing between 24 and 48 h of continuous data (e.g., ECG, PPG, arterial BP) recorded from patient monitors in the medical, surgical, and cardiac intensive care units of Boston's Beth Israel Hospital. Detailed clinical data accompany each record. ECG was measured on the chest, and PPG on the first (i.e., index) finger. An invasive catheter and BP sensor measured arterial BP in the pulmonary artery. All patients were hemodynamically unstable, and 31 yielded usable data. Institutional Review Board Supervision of the original data was representative of standard ICU practice by the Committee on Clinical Investigations (#2001P001699) that reviewed this research involving human subjects at the Beth Israel Hospital and is documented within Physionet.

### Theoretical rationale for feature selection

The relation between Pulse Pressure (PP), PTT and PWM or pulse wave contour is determined by the theory of the propagation of a Newtonian liquid through a long tube with blood having a specific viscosity and density flowing through this tube. Using the Moens-Korteweg equation and the Bramwell Hill equation for compliance, volume and pressure, Thambiraj et al.^33^ showed that PP is a function of PTT, pulse intensity ratio (PIR) and the Womersley number ($\propto$) which depends on the viscosity and density of blood. Using the well-known estimation for PP in Eq. (2) below,

2 $$PP=\frac{Stroke \; Volume}{Compliance},$$

And since stroke volume is proportional to the pre-ejection period (PEP), PP^33^ can be shown as in Eq. (3) below,

3 $$PP \propto \left(\frac{PEP}{PIR . PT{T}^{2}}\right) {\left.\left(1- \frac{0.56}{\sqrt{2}\propto }\right.\right)}^{-2}$$

Similarly, DBP, SBP and MAP, as indicated in Eq. (4) through Eq. (6) below, can be written as follows^33^,

4 $$DBP \propto \mathrm{ln} \left[\left(\frac{PEP}{PIR . PT{T}^{2}}\right) {\left.\left(1- \frac{0.56}{\sqrt{2}\propto }\right.\right)}^{-2} \right]$$

5 $$SBP=PP+DBP$$

6 $$MAP=\frac{1}{3} (2* DBP+1*SBP)$$

where DBP is diastolic BP, SBP is systolic BP, and MAP is Mean Arterial Pressure^34^.

PIR is approximated from the pulse wave contour, while PEP is the difference between the PAT and PTT. Since PEP does not exist during diastole, DBP can be derived through features associated with PAT and pulse wave contour. These are therefore included as input feature sets to our AI models. Since SBP is the sum of DBP and PP, it is determined by PEP in addition to PTT and PIR; and needs PEP as an additional input feature.

The main advantage of combining PAT and pulse wave contour is to ensure that all confounding factors are accounted for when making estimates of SBP and DBP. Dropping any of these factors will introduce variation in the estimation, thereby affecting the accuracy and variability of the estimate*.*

Therefore, for BP estimation in hemodynamically compromised patients from the MIMIC database, we chose a total of 12 input features. PAT and pulse rate were the two temporal components. Age and sex were the two demographic components. Eight morphology-based features^24^ were extracted from the PPG, consisting of BP cycle time (Tc), ejection or artery fill time (Ts, time in seconds from the start of the PPG waveform to the peak), artery emptying time (Td, time in seconds from the peak to the start of the next cycle), peak volume (Vp, volume depicted by the length from start of the PPG waveform to the peak), systolic volume (Vp times the ratio Ts/Tc), systolic volume differential (Vp divided by Ts), diastolic volume (Vp times Td/Tc) and diastolic volume differential (Vp divided by Td).

The MATLAB function findpeak () was used to extract the temporal components and morphology-based features to identify the peak times for the ECG R-waves and the peaks and valleys for the PPG. PAT was then calculated by averaging the interval difference between the peaks over five successive wave cycles, chosen via visual identification of artefact-free segments. The eight morphological features were extracted from these five PPG waveforms and averaged. Arterial systolic and diastolic pressures, measured using an intra-arterial catheter-based sensor inserted in the pulmonary artery, were similarly averaged over the five corresponding waveforms, forming the true systolic and diastolic values. Covering a broad range of hemodynamic data over the entire MIMIC database, up to five separate feature sets were extracted for each of the 31 subjects, giving a total of 126 separate feature datasets.

### Methods of analysis

Our hypothesis was that the use of optimized ML algorithms combined with critical multimodal features extracted from real-world data (RWD) sets could enable reliable and accurate estimation of continuous BP using cuffless devices.

Twenty-three healthy subject feature sets were used to confirm the feasibility of these ML methods for estimating SBP, DBP and MAP. Each subject's 23 feature data sets were used to train and independently test the ML algorithms using a leave-one-out methodology where training was performed on all but one datum point. The SBP, DBP, and MAP pressures were estimated on this remaining datum point. This process was repeated for all 23 combinations. We then estimated the average error bias and standard deviation over the 23 combinations. The "truth" output vector (i.e., label vector) consisted of the arterial systolic and diastolic pressures obtained using the manual auscultation method as outlined in the IEC 80601-2-30 standard^10^ for automated non-invasive sphygmomanometers (2018). "Truth" MAP was estimated as in Eq. (6).

The patient "test" dataset comprised 126 data sets from 31 hemodynamically compromised patients. Of the 126 datasets, 125 were used for training and one for testing (leave one out). This processing was repeated for all 126 combinations to estimate the average error bias and standard deviation for SBP, DBP, and MAP over the 126 combinations. We also used an 80:20 and a 70:30 split of the training and test dataset, and the training dataset was further split into a training and validation dataset. However, the leave-one-out approach was found to be more robust since it allowed the largest possible training set; therefore, data using this approach has been presented here.

The ML methods consisted of

1. Lasso (lasso command in MATLAB)—prediction based on a linear model using least squares regression coefficients on the feature data;
2. RF (Random Forest, fit ensemble command in MATLAB)—prediction based on a trained regression ensemble model that included boosting 100 regression trees;
3. SVM (Support Vector Machine, fitrsvm command in MATLAB)—prediction based on a support vector machine regression model using kernel functions;
4. ANN (Neural Network, new command in MATLAB)- prediction using the two-layer feed-forward network containing 20 neurons in the hidden layer, log sigmoid transfer function and the Levenberg–Marquardt backpropagation algorithm;
5. LSTM (Long Short Term Memory, LSTM layers and training options command in MATLAB) prediction using the "adam" or adaptive moment estimation optimizer using 140 hidden neurons, a sequential input layer and a fully connected output layer.

Given our data size and feature sets, we used Lasso, RF, SVM, and ANN to reduce system complexity. These algorithms are widely used classical machine learning algorithms used for prediction. Lasso is an extension of the linear regression model, while RF, SVM and ANN use alternate approaches based on non-linear decision-making that work well in predictive applications. We added a deep learning algorithm LSTM to explore how it would perform with a relatively minor dataset.

Initially, hyperparameter selection was evaluated for each ML method. Table 2 outlines the hyperparameters that were evaluated for each method. For Lasso, the elastic net hyperparameter ("alpha"), an estimate of the Lasso to ridge variance, varied between 0.6 and 0.8. In RF, the two significant hyperparameters consisted of the number of splits and the number of learning cycles, which varied in five combinations. Five optimal combinations of box constraint and kernel scale were evaluated for SVM. Finally, for the neural network and LSTM models, the number of epochs was varied for a given constant learning rate. The hyperparameters producing the lowest standard deviation BP estimation error (superscripts a, b and c and bolded in Table 2 below) were chosen for each model to estimate SBP, DBP and MAP.

**Table 2:** Hyperparameter selection.

Trial #	Lasso	RF		SVM		ANN	LSTM
	Alpha	Splits	Learning cycles	Box constraint	Kernel scale	Epochs	Epochs
1	0.6	**80**^**a**^	**300**^**a**^	0.001	0.31	500	500
2	0.65	100	400	0.006	0.7	1000	**1000**^**a**^^**,b**^
3	**0.7**^**a**^	**120**^**b**^	**500**^**b**^	0.1	0.65	**1500**^**a**^	1500
4	**0.75**^**b**^	140	600	1	0.48	2000	2000
5	0.8	160	700	**166**^**a**^^**,b**^	**0.82**^**a,b**^	**2500**^**b**^	2500

^a^Hyperparameters with the lowest systolic standard deviation (in bold).

^b^Hyperparameters with the lowest diastolic standard deviation (in bold).

The ‘learning rate’ was also one of the hyperparameters used in our evaluation in Table 2 above. However, after several iterations of the learning rate, all the algorithms were highly sensitive to the learning rate, thereby making the selection of the optimal learning rate relatively obvious, which is why it was not included as part of Table 2. The learning curves can be found in the Supplementary Information.

### Methods of statistical analysis

Statistical analysis on the healthy dataset (N = 126) consisted of computing the bias (mean error) and the standard deviation error along with the 95% confidence intervals for feasibility analysis.

Statistical analysis on the hemodynamically compromised dataset (N = 126) consisted of descriptive statistics and statistical comparisons between the five ML methods and three feature sets—combined, PAT and morphology. For each of the five ML algorithms applied to the patient data, we measured the bias (mean error) and standard deviation error between estimated SBP, DBP and MAP in comparison to the labelled data, as well as their 95% confidence intervals. This was repeated for the three feature sets. Two-way analysis of variance (ANOVA) was used to test for significant differences in *mean error* between the five ML methods and three feature sets. When significant differences were found, post hoc pairwise F-tests were performed. Levene’s tests were conducted on the test dataset to statistically compare the mean *absolute* error (a standard deviation measure) between the five ML methods and the three feature datasets. When significant differences were found without interaction, post hoc pairwise t-tests were performed. When interactions were found, we performed post hoc pairwise t-test comparisons of all factor combinations since the number of combinations was small. All post hoc analyses used Bonferroni-Holm correction for multiple comparisons.

## Results

Demographic information for the healthy and MIMIC subjects (hemodynamically compromised) are shown in Table 1. At least 40% of the subjects were female, and over 50% were above 40 years of age.

Table 3 shows the mean, standard deviation and 95% confidence interval errors for the healthy dataset (n = 23) using the leave-one-out method on all five ML methods and the PTT feature dataset. For each model except for LSTM, each bias error met the IEC 80601-2-30 (2018) standard of ≤ 5 mmHg. Only DBP for the RF and Lasso models additionally exhibited an error SD ≤ 8 mmHg, satisfying criteria 1 of the IEC 80601-2-30:2018 standard^10^. Both SBP and MAP had error SDs over 8 mmHg for all five algorithms.

**Table 3:** Mean (μ), standard deviation (SD) and 95% confidence interval (CI) for signed errors (mmHg) in estimating MAP, SPB and DBP for the healthy and hemodynamically compromised dataset.

	Lasso		Random forest		SVM		ANN		LSTM	
	μ ± SD	(95% CI)	μ ± SD	(95% CI)	μ ± SD	(95% CI)	μ ± SD	(95% CI)	μ ± SD	(95% CI)
Healthy subjects (n = 23 subjects):										
MAP	− 0.18 ± 11.51	(− 4.88, 4.53)	0.27 ± 9.51	(− 3.62, 4.16)	− 2.18 ± 9.26	(− 5.97, 1.60)	− 0.21 ± 10.15	(− 4.36, 3.94)	− 10.15 ± 10.61	(− 14.49, − 5.82)
SBP	1.77 ± 22.46	(− 7.41, 10.95)	− 0.65 ± 15.28	(− 6.89, 5.59)	3.44 ± 13.56	(− 2.10, 8.98)	1.27 ± 19.62	(− 6.75, 9.29)	10.63 ± 19.37	(2.71,18.55)
DBP	− 0.62 ± 7.64^a^	(− 3.74, 2.50)	− **0.08 ± 7.49**^**a**^	(− 3.14, 2.98)	1.56 ± 8.62	(− 1.96, 5.08)	− 0.31 ± 8.76	(− 3.89, 3.27)	9.92 ± 13.51	(4.40, 15.44)
Hemodynamically compromised patients (N = 126 records from 31 subjects) for combined feature dataset:										
MAP	0.03 ± 11.89	(− 2.05, 2.11)	0.76 ± 8.84	(− 0.78, 2.3)	0.75 ± 10.56	(− 1.09, 2.59)	1.17 ± 11.12	(− 0.77, 3.11)	2.25 ± 13.39	(− 0.09, 4.59)
SBP	− 0.14 ± 18.31	(− 3.34, 3.06)	1.38 ± 15.12	(− 1.26, 4.02)	0.43 ± 17.7	(− 2.66, 3.52)	1.65 ± 21.62	(− 2.13, 5.43)	3.78 ± 21.82	(− 0.03, 7.59)
DBP	0.03 ± 8.97	(− 1.53, 1.60)	**0.45 ± 7.53**^**a**^	(− 0.86, 1.76)	**0.91 ± 7.32**^**a**^	(− 0.37, 2.19)	− 0.93 ± 9.23	(− 2.54, 0.68)	1.49 ± 9.17	(− 0.11, 3.09)
Hemodynamically compromised patients for PAT dataset (N = 126 records from 31 subjects):										
MAP	0.00 ± 12.67	(− 2.21, 2.21)	− **0.08 ± 6.57**^**a**^	(− 1.23, 1.07)	0.95 ± 9.72	(− 0.74, 2.65)	− 0.26 ± 8.07	(− 1.67, 1.15)	2.32 ± 13.46	(− 0.03, 4.67)
SBP	0.00 ± 20.8	(− 3.63, 3.63)	− 0.52 ± 15.4	(− 3.21, 2.17)	0.63 ± 16.44	(− 2.24, 3.5)	− 1.52 ± 18.12	(− 4.68, 1.64)	4.13 ± 22.03	(0.28, 7.98)
DBP	0.00 ± 9.03	(− 1.58, 1.58)	**0.14 ± 4.51**^**a**^	(− 0.65, 0.93)	**1.09 ± 6.69**^**a**^	(− 0.08, 2.26)	**0.37 ± 5.92**^**a**^	(− 0.66, 1.40)	1.42 ± 9.18	(− 0.18, 3.02)
Hemodynamically compromised patients for morphology dataset (N = 126 records from 31 subjects):										
MAP	0.09 ± 12.46	(− 2.08, 2.27)	− 0.14 ± 8.55	(− 1.63, 1.35)	0.89 ± 10.11	(− 0.87, 2.66)	− 0.94 ± 11.41	(− 2.93, 1.05)	1.36 ± 13.1	(− 0.93, 3.65)
SBP	− 0.11 ± 18.83	(− 3.39, 3.18)	0.09 ± 17.51	(− 2.97, 3.15)	0.57 ± 17.24	(− 2.44, 3.58)	2.49 ± 24.22	(− 1.74, 6.72)	4.00 ± 21.99	(0.16, 7.84)
DBP	− 0.14 ± 8.8	(− 1.68, 1.40)	**0.05 ± 7.61**^**a**^	(− 1.28, 1.38)	**1.01 ± 7.35**^**a**^	(− 0.27, 2.30)	0.65 ± 11.55	(− 1.37, 2.67)	0.045 ± 8.66	(− 1.47, 1.56)

^a^Within 5 mm for bias and 8 mm for standard deviation (in bold).

**Figure 3:**
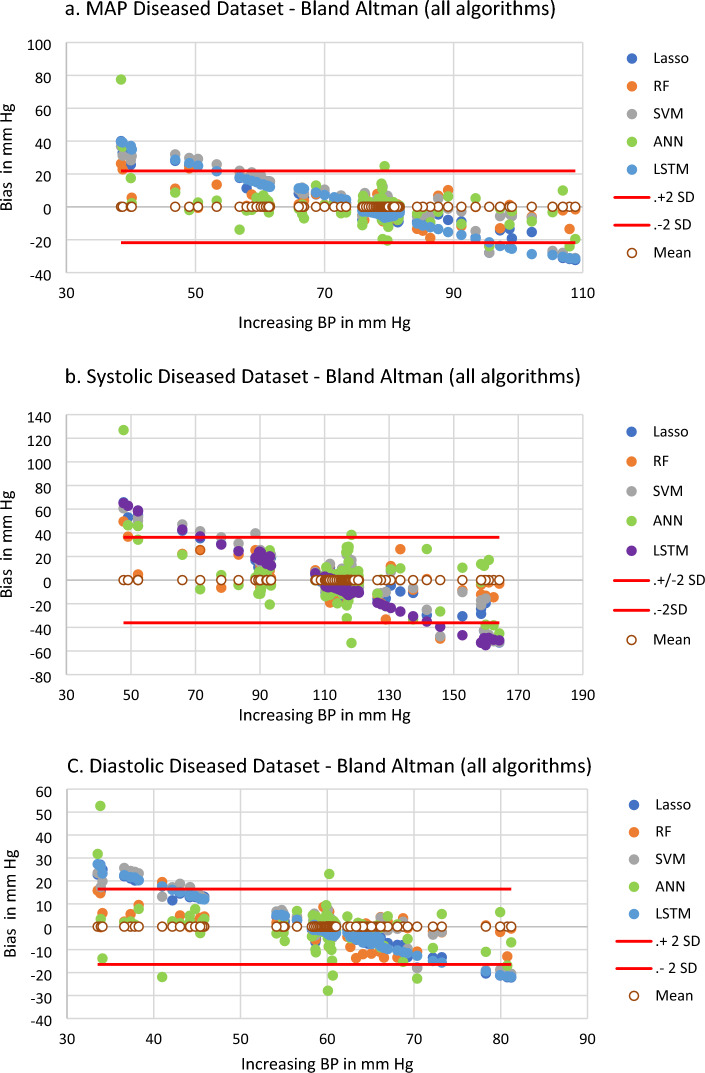
Estimated BP in the independent test dataset using combined feature datasets, hemodynamically compromised patients, n = 126. Lines show ± 2 standard deviations of all five algorithms. MAP comparison. b. SBP comparison. c. DBP comparison. Axis scales differ between plots.

Table 3 also shows the mean, standard deviation and 95% confidence interval errors for the hemodynamically compromised subjects (n = 31, 126 datasets) using the leave-one-out method on all five ML methods and all three feature datasets—combined, PAT and PWM or morphology. Every bias error met criterion 1 of the IEC 80601-2-30:2018 standard^10^, each being ≤ 5 mmHg. Only eight of 45 ML method-feature dataset combinations met the SD ≤ 8 mmHg standard, with no such models using the Lasso or LSTM methods. Seven of the eight models meeting the SD standard were for DBP. The lowest SD used the PAT dataset and estimated diastolic pressure using the RF model, giving a mean ± SD error of 0.14 ± 4.51 mmHg. Bland–Altman plots for the SBP, DBP and MAP using the combined feature set, leave-one-out methodology and for each of the ML methods are shown in Fig. 3.

RF performed better than ANN using the PAT dataset—the only dataset for which MAP error met criterion 1 of the IEC standard for the RF model. However, the SD difference between RF and ANN for this was only 1.5 mmHg. None of the model-feature dataset combinations for SBP fully met the IEC standard.

Table 4 presents the statistical comparison results for the *mean* errors. While several significant differences were reported, it is important to note that all model-feature dataset combinations met the IEC standard for bias or mean error. Hence, each poorer-performing combination still exhibited an acceptable error. Two-way ANOVAs (one per pressure) were used to compare differences in *means* of the estimation errors between the five ML methods and three feature sets in the hemodynamically compromised dataset. Table 4 shows a significant difference between the five ML methods, but not the feature datasets, without interaction for each pressure.

**Table 4:** Two-way analysis of variance F-test and p-value results for significant differences in mean errors for MAP, SBP and DBP using 5 ML methods and 3 feature sets in hemodynamically compromised subjects (N = 126).

Method	MAP	SBP	DBP
Feature	F (2) = 0.04, p = 0.96	F (2) = 0.06, p = 0.95	F (2) = 0.065, p = 0.94
Machine learning	F (4) = 4.88, p = 0.00065*	F (4) = 6.08, p = 0.000073*	F (4) = 3.58, p = 0.0065*
Interaction—feature and ML	F (2,4) = 0.05, p = 0.99	F (2,4) = 0.035, p = 0.99	F (2,4) = 0.11, p = 0.99

*Significant difference (in bold).

Post hoc paired comparisons were conducted only on the combined feature dataset (since no significant differences were found between the three feature datasets and their data are highly correlated) between the five ML algorithms, as shown in Table 5. Only 11 of 30 pairwise comparisons between methods demonstrated a statistically significant difference. The LSTM method never showed statistically lower error performance. The SVM method most frequently showed statistically lower error performance (8 of the 11 differences).

**Table 5:** Machine learning method—Post hoc statistical results to test for differences in mean errors for each of the machine learning methods using a combined feature dataset in hemodynamically compromised subjects (N = 126).

	RF	SVM	ANN	LSTM
Mean				
Lasso	NS	F (1) = 4.99, p = 0.007* (SVM)	NS	NS
RF		NS	NS	NS
SVM			NS	F (1) = 15.5, p = 0.000* (SVM)
ANN				NS
Systolic				
Lasso	NS	NS	NS	F (1) = 7.02, p = 0.001* (Lasso)
RF		F (1) = 5.36, p = 0.005* (RF)	NS	F (1) = 11.9, p = 0.001* (RF)
SVM			NS	F (1) = 15.5, p = 0.000* (SVM)
ANN				F (1) = 7.21, p = 0.007* (ANN)
Diastolic				
Lasso	NS	F (1) = 5.6, p = 0.004* (SVM)	NS	NS
RF		F (1) = 7.57, p = 0.001* (RF)	NS	NS
SVM			F (1) = 9.43, p = 0.002* (ANN)	F (1) = 9.05, p = 0.003*(SVM)
ANN				NS

Parentheses denote the method with the significantly *lower* mean error.

*Denotes a significant difference; NS indicates not significant.

Data are shown as F and p values.

Table 6 presents the statistical comparison results for the *absolute* errors (which are related to the error standard deviations). The statistical results confirm that both the Lasso and LSTM methods did not perform well. The LSTM method never exhibited lower errors than other methods, and the Lasso method only performed better than LSTM—but still not acceptable according to the IEC standard. Separately for each pressure, the difference in *absolute* error (*standard deviation*) between the five ML methods and three feature sets was investigated using Levene’s test, as seen in Table 6. A significant difference was found between the five ML methods for DBP, but not among the feature datasets, without interaction. For MAP and SBP, the respective Levene test found an interaction (Table 6). Thus, post hoc pairwise comparisons were conducted for each pressure between all combinations of the three feature datasets and five ML methods.

**Table 6:** Levene’s test results of significant differences in absolute error (standard deviation) for SBP, DBP and MAP in hemodynamically compromised subjects (N = 126 datasets from 31 subjects).

Method	MAP	SBP	DBP
Feature	F (2) = 2.50, p = 0.082	F (2) = 2.59, p = 0.075	F (2) = 2.47, p = 0.085
Machine learning	F (4) = 22. 9, p = 0.00*	F (4) = 20.92, p = 0.00*	F (4) = 16.3, p = 0.00*
Interaction—feature and ML	F (2,4) = 2.54, p = 0.0095*	F (2,4) = 2.81, p = 0.004*	F (2,4) = 1.89, p = 0.058

*Denotes a significant difference.

Data are shown as F and p values.

Table 7 shows post hoc paired comparison results using the three feature datasets between the five ML algorithms. Across all three pressures, 17 of 30 cells within Table 7 demonstrated a statistically significant difference. The LSTM method never showed statistically lower error performance. The RF method most frequently showed statistically lower error performance (9 of the 17 differences). For DBP, Table 7 shows that SVM performed statistically better than RF and ANN, while RF performed better than ANN based on *absolute* errors. However, Table 3 shows that the actual SD difference was < 2 mmHg between these methods for DBP using the combined features dataset—and the corresponding SD difference between RF and SVM was only 0.21 mmHg. Overall, our results seem to find these two methods somewhat equivalent in performance for DBP estimation. For MAP and SBP, RF performed significantly better than SVM and ANN based on *absolute* errors (standard deviation).

**Table 7:** Levene’s test post hoc results to test for significant differences in absolute error (standard deviation) for hemodynamically compromised subjects (N = 126 datasets from 31 subjects).

	RF	SVM	ANN	LSTM
Mean				
Lasso	p = 0.000* (RF) [combined]	NS	NS	NS
RF		p = 0.000* (RF) [combined]	p = 0.000* (RF) [combined] p = 0.000* (RF) [PAT]	p = 0.000* (RF) [combined]
SVM			NS	NS
ANN				p = 0.000* (ANN) [combined]
Systolic				
Lasso	p = 0.000* (RF) [combined]	NS	NS	p = 0.000* (Lasso) [combined]
RF		p = 0.000* (RF) [combined]	p = 0.000* (RF) [combined] p = 0.000* (RF) [PAT]	p = 0.000* (RF) [combined]
SVM			p = 0.002* (SVM) [morphology]	p = 0.000* (SVM) [combined]
ANN				p = 0.000* (ANN) [combined]
Diastolic				
Lasso	p = 0.000* (RF) [combined]	NS	NS	NS
RF		p = 0.000* (SVM) [combined]	p = 0.000* (RF) [combined] p = 0.000* (RF) [PAT]	p = 0.000* (RF) [combined]
SVM			NS	NS
ANN				NS

For MAP, DBP and SBP, the results are shown for each pairwise combination of the feature dataset and machine learning method.

*All p values denote a significant difference using the Bonferroni-Holm method; NS indicates not significant.

Data are shown as p values.

Parentheses denote the method with the significantly *lower* mean error. Square parentheses denote the feature dataset.

In summary, RF and SVM gave the best overall performance for this dataset.

## Discussion

Our goal was to present a theoretical framework and demonstrate how the use of optimized ML algorithms combined with critical multimodal features extracted from real-world data (RWD) sets could enable reliable and accurate estimation of continuous BP using cuffless devices. Our method evaluated various ML algorithms for estimating BP using critical multimodal features. We demonstrated the feasibility of our methodology using healthy subjects with normal BP values. We then tested our hypothesis on hemodynamically compromised (diseased) subjects having abnormal BP values.

Initially, we evaluated the hyperparameters for each ML algorithm. These parameters were varied in five different combinations. We then fixed the respective hyperparameters in the models using the one with the least standard deviation. We found that each of the model-feature dataset combinations produced a bias error ≤ 5 mmHg, which falls within the acceptability criterion 1 of the IEC/ANSI 80601-2-30 (2018) standard^10^. For DBP estimation, our results with the RF, SVM and ANN methods were also within the 8 mmHg SD limits of the BP performance standard (criterion 1, IEC 80601-2-30 Standard for BP monitoring, 2018). Our SBP and MAP estimates showed a higher variation compared to DBP, most likely due to the additional variation introduced by the inclusion of the pre-ejection period that increases the transit time of the pulse wave^16,35^. Only eight of 45 method-feature dataset combinations had an SD ≤ 8 mmHg, as required by the IEC standard. Of these combinations, none included the Lasso or LSTM models; hence, these models proved ineffective on these data. In addition, seven combinations included DBP, one included MAP and none included SBP. In summary, RF and SVM gave the best performance overall in terms of confidence intervals for this dataset. Choosing the best ML algorithm from a multitude of algorithms was found to significantly improve the accuracy and reliability of the estimation.

We used 126 datasets from 31 hemodynamically compromised patients. By increasing the number of subjects, application of our methodology might further reduce the bias and the variation to give better estimates of SBP, DBP, and MAP. The use of a larger dataset comprising over 2000 subjects may help reduce this variation. Subsequent research should therefore focus on the use of a greater number of subjects for inclusion in the training of the ML algorithms.

The key limitation of our approach has been the use of PAT instead of PTT as one of our input features in the hemodynamically compromised dataset. PEP has been suggested^16,35^ to present a significant limitation to SBP estimations that use PAT instead of PTT, as elaborated in our theoretical rationale in the “Methods” section^36 ^ above. Future work should use the PEP in addition to the PAT or the PTT, especially for SBP and MAP estimation. Another key limitation of our method is the long-term stability of our PPG-based approach. We have not evaluated the stability in patients over time or the time-period over which such a recalibration would be required. Future work should focus on estimating the period over which this method can give stable results before requiring a recalibration.

Using ANOVA and Levene’s test for error means and standard deviations, we found significant differences in the various ML algorithms but found no statistically significant differences amongst the multimodal feature datasets. An issue here might be the interplay between the number of fit parameters in a model and the available size of the training data set^37^. A rough guide to mitigate “overfitting” is for the training set size to be at least ten times that of the number of fit parameters^38^. However, with only 126 data sets in our study, this guidance was not met. In particular, the combined feature set necessarily had the largest number of fit parameters. While the increased number of parameters increased the available modeling detail, this increase may have been offset by the limited training size. Our use of leave-one-out cross-validation allowed the largest possible training set. In any case, future modeling with larger RWD datasets may find an advantage in using the combined feature set that includes both PAT, PEP and PWM measurements instead of relying on any one of these measurements alone.

Here, we have demonstrated how the use of optimized ML algorithms applied to carefully selected multimodal, high-quality features derived from large RWD sets could enable a platform for accurate and reliable estimation of continuous BP in a cuffless device. Future work should consider the application of more sophisticated algorithms to the selected features. Such algorithms could include applying multi-branch convolution and pooling operations to a large comprehensive dataset with a multiscale feature extraction module to obtain shallow and deep features and a multimodal fusion method that merges low-level detailed, and high-level features^36^. Li et al.^39^ elaborate on a Cov-Net architecture consisting of a feature learning module that extracts valuable information from the input feature sets and another feature fusion model set for further generating and merging multi-level feature maps. This approach should be explored on the different feature sets of demographic data, PAT or PTT, PEP and morphology.

Various other techniques, such as deep belief networks or DBNs and Deep Reinforcement learning or DRL, have been suggested by Li et al.^40^ for point-of-care testing systems. Such methods could be utilized in future work on a large dataset with adequate feature resolution. While the algorithms we used utilized well-known single and double-order optimizers such as the ADAM optimizer, future work could include recent, alternate optimization techniques. Some such optimization techniques include the planet optimization algorithm^41^, which uses an optimizer based on stochastic decisions and surrounding exploration, the Runga Kutta method-based RUN optimization^42^, which effectively implements and balances the exploration and exploitation in the search phase and the Hunger Games search^43^, which designs and employs an adaptive weight based on the concept of hunger on each search step.

Our conclusion clearly indicates that advances in sensor technology, extraction of high quality features from large RWD datasets and use of optimized ML algorithms will help unlock the great promise of continuous cuffless BP estimation, with profound clinical implications. This will lead to reliable and accurate non-invasive cuffless monitoring of continuous BP at home, measurement of diurnal variations, night time variations and night-to-day variations. It will also allow us to see the effect of various environmental and physiological factors on continuous BP and other critical features. Additional research needs to be conducted to determine if there are significant correlations between diurnal and nocturnal variation in BP as well as short-term variation in BP and various types of diseases. Other data features extracted from high quality continuous BP monitoring combined with optimized ML algorithms may also elucidate novel markers for improving diagnosis of diseases or improving clinical management of patients. Continuous, ambulatory BP monitoring can potentially provide additional data that cannot be gleaned from intermittent and sporadic BP measurements. The ability to perform reliable and accurate continuous BP measurements and collect larger RWD data sets on diurnal variation and short-term variability will help provide fresh clinical insights and new tools for the early detection of diseases^9,12^ like cardiac morbidity, chronic renal failure, malignant and secondary hypertension, pre-eclampsia and autonomic neuropathy (Supplementary Information).

## Supplementary Information

Supplementary Information.

## Data Availability

The data are available upon request from Rajesh S Kasbekar, rkasbekar@gmail.com, Department of Biomedical Engineering, Worcester Polytechnic Institute, Worcester, MA, USA.

## Code availability

Available upon request from Rajesh S Kasbekar, rkasbekar@gmail.com, Department of Biomedical Engineering, Worcester Polytechnic Institute, Worcester, MA, USA.
