# Supplementary material for: Optimizing the input feature sets and machine learning algorithms for reliable and accurate estimation of continuous, cuffless blood pressure
Source: Sci Rep. 2023 May 12;13:7750. doi: 10.1038/s41598-023-34677-9 (PMC10181996; doi:10.1038/s41598-023-34677-9)
Supplement: Supplementary file 1 — Supplementary Information. [file 41598_2023_34677_MOESM1_ESM.docx]

**SUPPLEMENTARY INFORMATION**

**Optimizing the input feature sets and machine learning algorithms for reliable and accurate estimation of continuous, cuffless blood pressure.**

**Rajesh S. Kasbekar^*1^, Songbai Ji^1^, Edward A. Clancy^1,2^, Anita Goel^3^**

^1^Department of Biomedical Engineering, Worcester Polytechnic Institute (WPI), Worcester, MA, USA

^2^Department of Electrical and Computer Engineering, Worcester Polytechnic Institute (WPI), Worcester, MA, USA

^3^Nanobiosym Research Institute, Nanobiosym, Inc. and Department of Physics, Harvard University Cambridge, MA, USA

***Corresponding author**

Rajesh S. Kasbekar

E-mail: rkasbekar@gmail.com

**ORCID**

Orcid ID (Rajesh Kasbekar): 0000-0002-7332-0406

**SUPPLEMENTARY NOTE 1**

1. TRANING AND VALIDATION CURVES FOR MACHINE LEARNING AND DEEP LEARNING ALGORITHMS

The figures below show the training and validation curves for each of the four machine learning and one deep learning algorithms. The curves are for both the diastolic and systolic blood pressures (DBP and SBP), but not for the mean arterial pressure (MAP), since MAP is derived from the SBP and DBP using equation (6) from the manuscript.

***Training curves***

***Diastolic BP***

*Figure S1. Training Curve for Diastolic Pressure - Lasso*

*Figure S2. Training Curve for Diastolic Pressure – Random Forest*

*Figure S3. Training Curve for Diastolic Pressure – Support Vector Machine*

***Training curves***

***Diastolic BP***

*Figure S4. Training Curve for Diastolic Pressure – Artificial Neural Network*

*Figure S5. Training Curve for Diastolic Pressure – Long Short Term Memory*

***Training curves***

***Systolic BP***

*Figure S6. Training Curve for Systolic Pressure - Lasso*

*Figure S7. Training Curve for Systolic Pressure – Random Forest*

*Figure S8. Training Curve for Systolic Pressure – Support Vector Machine*

***Training curves***

***Systolic BP***

*Figure S9. Training Curve for Diastolic Pressure – Artificial Neural Network*

*Figure S10. Training Curve for Diastolic Pressure – Long Short Term Memory*

***Validation curves***

***Diastolic BP***

*Figure S11. Validation Curve for Diastolic Pressure - Lasso*

*Figure S12. Validation Curve for Diastolic Pressure – Random Forest*

*Figure S13. Validation Curve for Diastolic Pressure – Support Vector Machine*

***Validation curves***

***Diastolic BP***

*Figure S14. Validation Curve for Diastolic Pressure – Artificial Neural Network*

*Figure S15. Training Curve for Diastolic Pressure – Long Short Term Memory*

***Validation curves***

***Systolic BP***

*Figure S16. Validation Curve for Systolic Pressure - Lasso*

*Figure S17. Validation Curve for Systolic Pressure – Random Forest*

*Figure S18. Validation Curve for Systolic Pressure – Support Vector Machine*

***Validation curves***

***Systolic BP***

*Figure S19. Validation Curve for Systolic Pressure – Artificial Neural Network*

*Figure S20. Validation Curve for Systolic Pressure – Long Short term Memory*

**SUPPLEMENTARY NOTE 2**

1. HYPERTENSION AND METHODS OF MEASURING BLOOD PRESSURE

Hypertension, defined as BP of 140/90 mm Hg or greater, as well as the use of antihypertensive medications, remains the leading risk factor for death and quality of life in high- and low-income countries. It affects approximately twenty percent of the world’s population or approximately 1.28 billion people at some time in their lifespan. This number is expected to reach 1.5 billion by 2025. The residual lifetime risk (defined as lifetime cumulative incidence not adjusted for competing causes of mortality) for developing hypertension in middle-aged and elderly individuals is 90%, indicating a huge public health burden to any healthcare system in the world^2^. Detection, monitoring and control, however, are inadequate with only 46% of individuals with hypertension having their condition under control^3^. The complications of hypertension are responsible for premature deaths worldwide^1^.

Traditionally, intermittent BP is estimated using a cuff-based monitor. However, cuff-based methods have proven to be a barrier to widespread use of BP measurement due to discomfort associated with measurements and the bulky equipment that is needed. A simple and user-friendly device that also measures continuous diurnal BP would better enable the management of hypertension. Continuous or ambulatory BP monitoring would also provide additional information than the information that is captured from clinical or home-use BP measurements^4^. Due to the limitations of methods requiring external pressure to the arteries in the arm or wrist, techniques for continuous BP monitoring have become popular.

An automated BP cuff employing oscillometry can be used to measure BP in clinical and home settings. A pressure sensor in the cuff measures arterial pulsations during cuff deflation/inflation, and the pulse amplitudes are used to empirically derive systolic and diastolic BP. These algorithms are specific to the device and the cuff. If the pulse amplitude is weak, it can result in inaccurate readings, such as in obese people or for someone who has atheroschlerotic plaques^5^. In addition, the application of pressure during cuff inflation/deflation can alter BP, resulting in a corrupted measurement.

Other techniques used for intermittent BP monitoring include the ultrasound technique or the Penaz finger cuff method. In the ultrasound method, an ultrasound transmitter and receiver placed under a cuff detect a Doppler phase shift during the diastolic phase^6^. The finger cuff method was first developed by Penaz^7^ and works on the principle of loading the arterial wall. In this method, arterial pulsation, as detected by a photoplethysmogram (PPG), is used to apply cuff pressure to the finger to keep the cardiac output constant. The resulting oscillations resemble intraarterial pressure waves, and this method gives systolic and diastolic pressure that is calibrated using an arm cuff. This method can be used to measure continuous BP but is expensive, inconvenient, highly variable based on its location as well as thermal characteristics of the finger, and limited in its application for hypotensive patients such that it is not suitable for consumer use^8^.

**^REFERENCES^**

^1^*WHO Q & A’s on hypertension*. <https://www.who.int/news-room/fact-sheets/detail/hypertension>. (2021)

^2^Vasan, R. S. et al, Residual lifetime risk for developing hypertension in middle-aged women and men: the framingham heart study. *JAMA* **287**:1003–1010. <https://doi.org/10.1001/jama.287.8.1003>. (2002).

^3^Merai, R. et al. CDC grand rounds: a public health approach to detect and control hypertension. *MMWR Morb Mortal Wkly Rep* **65**:1261–1264[. https://doi.org/10.15585/mmwr.mm6545a3](file:///C:\Keydisk\PhD_2022\Final%20Publication%20BP%20-%20Cuffless\Publication%20Paper\Scientific%20Reports\.%20https:\doi.org\10.15585\mmwr.mm6545a3). (2016)

^4^Bard, D. M., Joseph. J, I., Van Helmond, N. Cuff-less methods for blood pressure telemonitoring. *Front Cardiovasc Med* **6**:40. <https://doi.org/10.3389/fcvm.2019.00040>. (2019)

^5^Forouzanfar, M. et al. Coefficient-free blood pressure estimation based on pulse transit time-cuff pressure dependence. *IEEE Trans Biomed Eng* **60**:1814–1824. <https://doi.org/10.1109/tbme.2013.2243148>. (2013)

^6^Zakrzewski, A.M., Anthony, B.W., Arterial blood pressure estimation using ultrasound: clinical results on

healthy volunteers and a medicated hypertensive volunteer. *Annu Int Conf IEEE Eng Med Biol Soc* **2017**:2154

–2157. <https://doi.org/10.1109/embc.2017.8037281>. (2017)

^7^Penaz, J, Criteria for set point estimation in the volume clamp method of blood pressure measurement.

*Physiol Res* **41**:5–10. (1992)

^8^Ward, M., Langton, J.A., Blood pressure measurement. *Contin Educ Anaesth Crit Care Pain* **7:**122–126.

<https://doi.org/10.1093/bjaceaccp/mkm022>. (2007)
